# Parecoxib is neuroprotective in spontaneously hypertensive rats after transient middle cerebral artery occlusion: a divided treatment response?

**DOI:** 10.1186/1742-2094-3-31

**Published:** 2006-12-06

**Authors:** Jesper Kelsen, Katrine Kjær, Gang Chen, Michael Pedersen, Lisbeth Røhl, Jørgen Frøkiær, Søren Nielsen, Jens R Nyengaard, Lars Christian B Rønn

**Affiliations:** 1 The Water and Salt Research Centre, University of Aarhus, DK-8000 Aarhus C, Denmark; 2 Department of Neurosurgery NK, University Hospital of Aarhus, Noerrebrogade 44, DK-8000 Aarhus C, Denmark; 3 Institute of Clinical Medicine, University Hospital of Aarhus, Brendstrupgaardsvej 100, DK-8200 Aarhus N, Denmark; 4 NEUROSEARCH A/S, Pederstrupvej 93, DK-2750 Ballerup, Denmark; 5 MR Research Centre, University Hospital of Aarhus, Brendstrupgaardsvej 100, DK-8200 Aarhus N, Denmark; 6 Department of Radiology, University Hospital of Aarhus, Noerrebrogade 44, DK-8000 Aarhus C, Denmark; 7 Institute of Anatomy, University of Aarhus, DK-8000 Aarhus C, Denmark; 8 Stereology and EM Research Laboratory and MIND Center, University of Aarhus, DK-8000 Aarhus C, Denmark

## Abstract

**Background:**

Anti-inflammatory treatment affects ischemic damage and neurogenesis in rodent models of cerebral ischemia. We investigated the potential benefit of COX-2 inhibition with parecoxib in spontaneously hypertensive rats (SHRs) subjected to transient middle cerebral artery occlusion (tMCAo).

**Methods:**

Sixty-four male SHRs were randomized to 90 min of intraluminal tMCAo or sham surgery. Parecoxib (10 mg/kg) or isotonic saline was administered intraperitoneally (IP) during the procedure, and twice daily thereafter. Nineteen animals were euthanized after 24 hours, and each hemisphere was examined for mRNA expression of pro-inflammatory cytokines and COX enzymes by quantitative RT-PCR. Twenty-three tMCAo animals were studied with diffusion and T_2 _weighted MRI within the first 24 hours, and ten of the SHRs underwent follow-up MRI six days later. Thirty-three SHRs were given 5-bromo-2'-deoxy-uridine (BrdU) twice daily on Day 4 to 7 after tMCAo. Animals were euthanized on Day 8 and the brains were studied with free-floating immunohistochemistry for activated microglia (ED-1), hippocampal granule cell BrdU incorporation, and neuronal nuclei (NeuN). Infarct volume estimation was done using the 2D nucleator and Cavalieri principle on NeuN-stained coronal brain sections. The total number of BrdU^+ ^cells in the dentate gyrus (DG) of the hippocampus was estimated using the optical fractionator.

**Results:**

We found a significant reduction in infarct volume in parecoxib treated animals one week after tMCAo (p < 0.03). Cortical ADC values in the parecoxib group were markedly less increased on Day 8 (p < 0.01). Interestingly, the parecoxib treated rats were segregated into two subgroups, suggesting a responder vs. non-responder phenomenon. We found indications of mRNA up-regulation of IL-1β, IL-6, TNF-α and COX-2, whereas COX-1 remained unaffected. Hippocampal granule cell BrdU incorporation was not affected by parecoxib treatment. Presence of ED-1^+ ^activated microglia in the hippocampus was related to an increase in BrdU uptake in the DG.

**Conclusion:**

IP parecoxib administration during tMCAo was neuroprotective, as evidenced by a large reduction in mean infarct volume and a lower cortical ADC increment. Increased pro-inflammatory cytokine mRNA levels and hippocampal granule cell BrdU incorporation remained unaffected.

## Background

Ischemic stroke is one of largest socioeconomic challenges in the health care systems of developed countries due to the large number of patients who are left disabled [1]. Apart from acute thrombolysis within the first three to six hours after onset of stroke symptoms, efficient treatment options are still lacking.

The importance of the cyclooxygenase 2 (COX-2) enzyme in ischemic brain injury has been emphasized by Iadecola et al. [2-4]. Several groups reported beneficial effects of COX-2 inhibition with wide therapeutic time windows in *in vitro *studies of glutamate-mediated cell death [5] as well as in different models of experimental brain ischemia [6-10], hemorrhage [11], and traumatic brain injury [12]. However, in September 2004 rofecoxib (Vioxx^®^) was voluntarily withdrawn by Merck because it had severe cardiovascular side effects after chronic administration [13]. Still, blockage of the COX-2 enzyme expressed on ischemic neurons and downstream effectors of COX-2 neurotoxicity remains an intriguing target in the reduction of glutamate exitotoxicity [14,15].

Parecoxib (Dynastat^®^) is a second generation COX-2 inhibitor, and registered as the only COX-2 inhibitor for intravenous (IV) administration. It is a pro-drug and hydrolyzed to the active metabolite valdecoxib. The ability of valdecoxib and other COX-2 inhibitors to cross the blood brain barrier (BBB) has been demonstrated in human studies [16]. Unfortunately, clinical trials with parecoxib and valdecoxib revealed the same adverse cardiovascular effects in high-risk patient populations [17]. Nevertheless, parecoxib does not increase the risk of myocardial infarction or stroke in low-risk populations referred to non-cardiac procedures [18]. To our knowledge, this is the first report addressing the effects of parecoxib in an experimental model of focal brain ischemia [19]. In addition we studied a possible drug effect on hippocampal granule cell BrdU incorporation as a measure for post-injury neuronal precursor cell (NPC) proliferation. Since neurogenesis following brain injury is one of the most encouraging endogenous repair mechanisms in the adult brain [20-23].

The aims of the present study were to investigate the effect of parecoxib treatment in spontaneously hypertensive rats (SHRs) subjected to transient middle cerebral artery occlusion (tMCAo) by determining: (1) messenger ribonucleic acid (mRNA) levels of key pro-inflammatory cytokines in brain tissue 24 hours after tMCAo, (2) apparent diffusion coefficient (ADC) values obtained from diffusion weighted imaging (DWI) 24 hours and one week after ischemic brain injury, (3) NPC proliferation in the dentate gyrus (DG) of the hippocampus one week after surgery, and (4) infarct volume estimated on immunohistochemically stained tissue sections one week after tMCAo.

## Methods

All male 14–16-week-old SHRs were purchased from Taconic (Germantown, NY 12526, USA) and housed in cages of two with free access to water and standard chow for laboratory rodents. The animals were kept in a twelve-hour day:night cycle and checked daily by professional staff. The experimental protocol was approved by The Animal Experiments Inspectorate (license no. 2003/561-702) under the Danish Ministry of Justice, and it fulfilled the requirements according to the European Community Council's Directive of November 24^th ^1986 (86/609/EEC).

### Study design

The current study was carried out at two different institutions. Animals (n = 27) subjected to magnetic resonance imaging (MRI) were studied at the Institute of Clinical Medicine (University Hospital of Aarhus, DK-8200 Aarhus N, Denmark), whereas the rest (n = 37) were operated at NEUROSEARCH A/S (DK-2750 Ballerup, Denmark). However, all animals were subjected to the same regimen and randomized to one of the following four groups: I. tMCAo + parecoxib intraperitoneally (IP) (n = 21); II. tMCAo + saline IP (n = 21); III. Sham + parecoxib (n = 8); and IV. Sham + saline (n = 9). Exclusion criteria were spontaneous death (n = 3); subarachnoid hemorrhage (n = 1); and missing ED-1 immunohistochemical positivity in animals subjected to tMCAo (n = 1).

In the first part of the study, animals (n = 19) were euthanized after 24 hours to examine the effect of parecoxib treatment on the mRNA level of Interleukin (IL)-1β, IL-6, tumor necrosis factor alpha (TNF-α), cyclooxygenase (COX)-1 and COX-2. Seven tMCAo animals only underwent MRI after 24 hours. In the second part of the study the remaining animals (n = 33) were subjected to subsequent injections of the thymidine analog, 5-bromo-2'-deoxy-uridine (BrdU) and euthanized after one week to study NPC proliferation in the molecular layer of the DG in the hippocampus by immunohistochemistry.

### Anesthesia protocol

**Figure 1:**
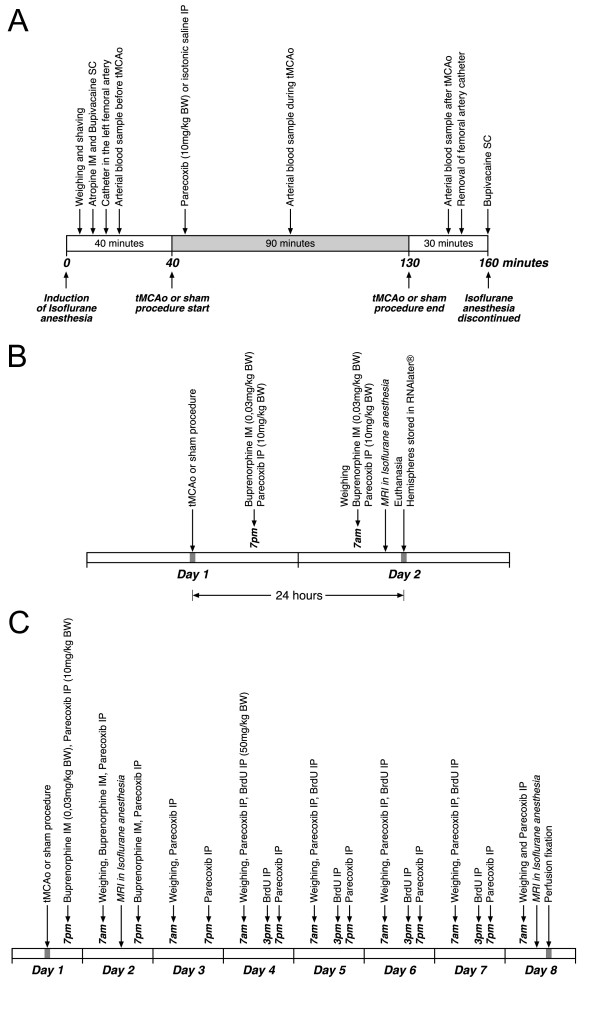
**Schematic maps of animal experiments**. The course of tMCAo and sham surgery is shown in **1A**. Note that all animals were anesthetized nearly 160 minutes while they underwent surgery. Parecoxib (10 mg/kg BW) or isotonic saline was administered IP within the first five minutes after the start of tMCAo or sham. **1B **illustrates steps in the qRT-PCR part of the study. Only half of the animals subjected to tMCAo underwent MRI prior to euthanasia. **1C **shows complete drug administration plan in the neurogenesis part. Buprenorphine (0.03 mg/kg BW) was given IM as a pain killer for the first two days twice daily. Parecoxib (10 mg/kg BW) or isotonic saline was injected IP twice daily throughout the investigation period. Finally, BrdU (50 mg/kg BW) was administered IP at 7 am and 3 pm on Day 4 to Day 7. Six tMCAo animals randomized to parecoxib treatment and four SHRs receiving isotonic saline commenced MRI on both Day 2 and Day 8.

Anesthesia induction was accomplished within two minutes in a chamber filled with 5% isoflurane (Baxter Isoflurane, Baxter Medical) in a 35/65% oxygen (O_2_) and nitrous oxide (N_2_O) atmosphere. Following weighing and shaving, the animals were placed in supine position on a heating pad and allowed to breathe spontaneously through a facemask. Isoflurane was decreased to 1.0–1.5% and administered continuously in the O_2_/N_2_O mixture at a flow rate of 1 L/min. The depth of anesthesia was assessed with toe pinching and on the basis of arterial blood gas parameters. An intramuscular (IM) injection of atropine (Atropin SAD, 0.05 mg/kg BW) was given to reduce mucus production during anesthesia. The incision sites were infiltrated with a subcutaneous injection of bupivacaine (Bupivacain SAD, 2.5 mg/ml) (Figure 1A).

Post surgery all animals were allowed to recover from anesthesia by inhaling 100% O_2 _until they regained consciousness. Buprenorphine (Temgesic^® ^Schering-Ploug, 0.03 mg/kg BW) was administered IM twice daily for the first two days as a post-surgical painkiller (Figure 1B and 1C).

### Monitoring of physiological parameters

A BD Neoflon™ (Becton Dickinson, Sweden) was inserted into the left femoral artery (FA) within the first ten minutes after induction of anesthesia and kept throughout surgery. Arterial blood samples were withdrawn before, during, and after the 90 minutes of tMCAo or sham surgery. pH, pCO_2_, and pO_2 _were measured immediately with an ABL500 or ABL615 blood gas analyzer (Radiometer, Copenhagen, Denmark). Hemoglobin and glucose were measured on HemoCue Photometers (HemoCue AB, Ängelholm, Sweden) or the ABL615.

**Figure 2:**
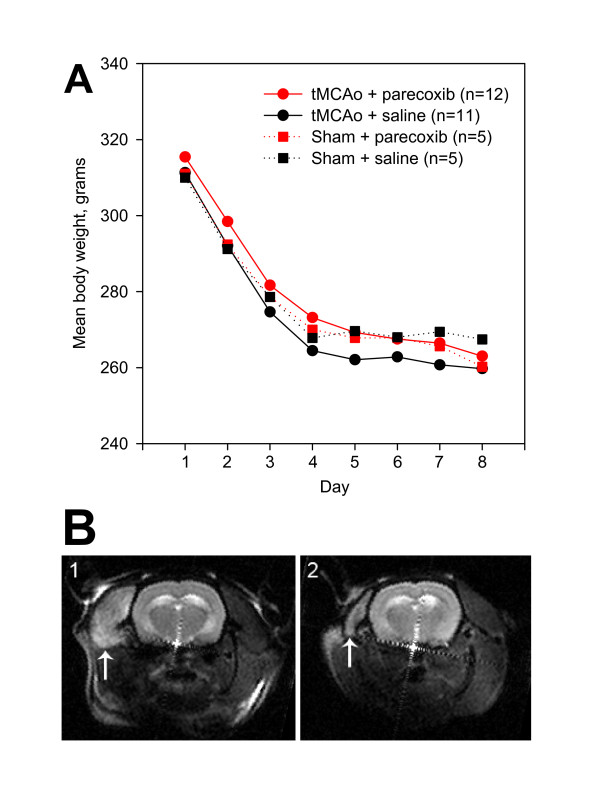
**Development in body weight after tMCAo or sham**. **2A **shows the development in the mean body weight after surgery for all four groups. Note the rapid decrease in body weight within the first four days where all animals lost around 15% of their preoperative body weight. black ■: sham + saline; red ■: sham + parecoxib; black ●: tMCAo + saline; and red ●: tMCAo + parecoxib. **2B **depicts the DWI of an animal obtained 24 hours (1) and one week after surgery (2). Although this animal apparently had no ischemic brain injury, there is a clear signal enhancement of the temporal muscle (white arrow) on the right side (**B1**). The signal changes are consistent with severe ischemia due to ECA ligation. Note the involution of the temporal muscle (white arrow) after one week (**B2**).

A PowerLab SP8 (ADInstruments, Castle Hill, NSW, Australia) was connected to a Bridge Amplifier that measured the middle arterial blood pressure (MABP) via a physiological pressure transducer (Capto SP 844, Memscap AS, Norway). The heart rate (HR) was determined from the systolic peeks on the arterial pressure curve by Chart 5 software version 5.1.1 (ADInstruments, Castle Hill, NSW, Australia). A rectal probe was coupled to a feed-back regulated heating pad system (Homeothermic Blanket Control Unit, Harvard Apparatus, Holliston, MA, USA) that kept the core temperature around 37.5°C. The animals were weighted daily to follow the post-surgical development in body weight (Figure 2A).

### Transient middle cerebral artery occlusion (tMCAo)

The right common carotid artery (CCA) was isolated through a small midline incision in the neck region. The vagus nerve was identified and carefully spared from surgical trauma. The right occipital artery (OA) and pterygopalatine artery (PA) were permanently ligated to assure that the filament was not trapped in wrong side branches. The superior thyroid artery (STA) was coagulated and transected to mobilize the external carotid artery (ECA). The ECA was ligated where it branches into the lingual (LA) and the maxillary artery (MA). Distal to the ligature the LA and MA were coagulated and cut. A small arteriotomy was made in the ECA stump and a filament with a rounded tip was introduced and maneuvered into the internal carotid artery (ICA) and advanced 22 mm beyond the carotid bifurcation. During the entire ischemic challenge, the right CCA was clamped to diminish blood flow. The intraluminal filament blocked the right MCA origin for 90 minutes. After withdrawal of the filament, the ECA stump was ligated and the CCA clamp removed. Reperfusion of the ICA was observed before wound closure. Animals in the two sham groups were subjected to exactly the same regimen, except that the filament was only advanced to the bifurcation of the PA and the ICA.

### Drug administration

Parecoxib (Dynastat^® ^Pfizer, 10 mg/kg BW) or an equivalent volume of isotonic saline was injected IP, within the first five minutes after the animals were randomized into one of the four groups. The parecoxib dosage was determined based on previous studies where COX-2 inhibitors were proven to be neuroprotective after experimental brain injury [8,11,12]. As specified in Figure 1B and 1C, parecoxib or isotonic saline were administered twice daily at 7 am and 7 pm.

### Magnetic resonance imaging (MRI)

Twenty-three SHRs subjected to tMCAo underwent DWI and T_2_WI in general anesthesia 24 hours after surgery. Ten of these animals went through a similar MRI sequence one week after tMCAo. Isoflurane anesthesia was induced in all SHRs as described above. The animals were oro-tracheally intubated and ventilated mechanically with a 1–2% isoflurane mixture during the MRI protocol. The head of the animals was positioned in a home-built surface radiofrequency receiver coil that fits into a 7-Tesla horizontal bore MR magnet (Oxford Instruments, Oxford, UK) equipped with a 12.5 G/cm gradient system (Tesla Engineering, West Sussex, UK). The magnet was interfaced to a Unity Inova console (Varian, Palo Alto, CA, USA).

DWI was performed using a spin-echo diffusion-sensitive imaging sequence with the following parameters: TR = 1.2 s, TE = 0.05 s, FOV = 4 × 4 cm^2^, slice thickness = 2 mm, interslice distance = 0 mm and data matrix = 256 × 256 pixels. Diffusion gradients equivalent to b values of 0 and 1401 × 10^-3 ^s/mm^2 ^(denoted as b_1 _and b_2_) were employed. T_2_-weighted imaging was carried out as a spin-echo multi-slice imaging sequence with the following acquisition parameters: TR = 1.5 s, TE = 0.05 s, FOV = 4 × 4 cm^2^, slice thickness = 2 mm, interslice distance = 0 mm and data matrix = 256 × 256 pixels. The ADC was estimated from the obtained signal intensity acquired with the two different b-values, S_1 _and S_2_, respectively:

ADC=1000×ln⁡[(S1/S2)(b2−b1)]
 MathType@MTEF@5@5@+=feaafiart1ev1aaatCvAUfKttLearuWrP9MDH5MBPbIqV92AaeXatLxBI9gBaebbnrfifHhDYfgasaacH8akY=wiFfYdH8Gipec8Eeeu0xXdbba9frFj0=OqFfea0dXdd9vqai=hGuQ8kuc9pgc9s8qqaq=dirpe0xb9q8qiLsFr0=vr0=vr0dc8meaabaqaciaacaGaaeqabaqabeGadaaakeaacqqGbbqqcqqGebarcqqGdbWqcqGH9aqpcqaIXaqmcqaIWaamcqaIWaamcqaIWaamcqGHxdaTcyGGSbaBcqGGUbGBdaWadaqaamaalaaabaGaeiikaGIaem4uam1aaSbaaSqaaiabigdaXaqabaGccqGGVaWlcqWGtbWudaWgaaWcbaGaeGOmaidabeaakiabcMcaPaqaaiabcIcaOiabdkgaInaaBaaaleaacqaIYaGmaeqaaOGaeyOeI0IaemOyai2aaSbaaSqaaiabigdaXaqabaGccqGGPaqkaaaacaGLBbGaayzxaaaaaa@4A3B@

Image post-processing of ADC maps was primarily done using the freeware ImageJ 1.34s [24]. Calculation of ADC was performed in a pixel-by-pixel basis. The cortex and subcortical area were delineated in the ischemic and contralateral hemispheres on calculated ADC maps. The ADC maps shown in Figure 7 were processed with Mistar software (Apollo Imaging Technology, Melbourne, Australia).

### Quantitative reverse transcriptase polymerase chain reaction (qRT-PCR)

Around 24 hours after the tMCAo or sham procedure ended, animals randomized to the qRT-PCR part of our study were decapitated in deep isoflurane anesthesia. The forebrain was divided into hemispheres and stored in RNAlater^® ^(Qiagen GmbH, Hilden, Germany) at 4°C until total RNA was extracted by means of an RNeasy Maxi Kit (Qiagen GmbH, Hilden, Germany). RNA preparations were treated with DNase I (Sigma-Aldrich, St. Louis, MO, USA) and verified to be DNA-free by PCR using rat β-actin specific primers (Table 1). First-strand cDNA was synthesized from 1 μg total RNA with a Oligo(dT)_20 _using SuperScript™ III First-Stand Synthesis System for RT-PCR (Invitrogen, Carlsbad, CA, USA) according to the manufacturer's instructions. qPCR for COX-1, COX-2, TNF-α, IL-1β, IL-6 and the house-keeping gene β-actin was carried out using 2 μl cDNA and Platinum^® ^SYBR^® ^Green qPCR SuperMIX UDG (Invitrogen, Carlsbad, CA, USA). Primers were designed with the open source software PerlPrimer [25] (Table 1).

**Table 1:** Primer sequences used for qRT-PCR.

**Gene**	**Forward primer (5' → 3')**	**Reverse primer (5' → 3')**	**Accession no**.
**COX-1**	GTACTATCCCTGAGATCTGGAC	TGAGTACTTCTCGGATGAAGG	S67721
**COX-2**	TGAGATACGTGTTGACGTCC	TTCCTTATTTCCTTTCACACCC	S67722
**TNF-α**	CTCTTCTCATTCCTGCTCGT	GAGAAGATGATCTGAGTGTGAG	AJ002278
**IL-1β**	CATAAGCCAACAAGTGGTATTCTC	TGTTTGGGATCCACACTCTC	NM_031512
**IL-6**	CAGGGAGATCTTGGAAATGAG	GGCAAATTTCCTGGTTATATCC	NM_012589
**β-actin**	TGACGGTCAGGTCATCACTATC	TGACGGTCAGGTCATCACTATC	NM_031144

The qPCR was run in triplicates using the DNA Engine OPTICON™ (MJ Research, Boston, MA, USA) and the cycling program was conducted as follows: 50°C for 2 min, 95°C for 2 min and subsequently forty-five cycles of 95°C for 15 s, 60°C for 30 s, and 72°C for 30 s. Products were electrophoresed to confirm specificity of the reactions. Quantification was performed by Opticon Monitor Analysis Software version 1.4. (MJ Research, Boston, MA, USA).

### BrdU labeling of neuronal precursor cell proliferation

The NPC proliferation one week after transient brain ischemia or sham was assessed using IP administration of the thymidine analog, BrdU. The BrdU dosage was 50 mg/kg BW (Sigma-Aldrich, St. Louis, MO, USA) twice daily on Day 4 to Day 7. The proliferation marker was given at 7 am and 3 pm to assure that proliferating cells were in the S phase of the mitotic cell cycle (Figure 1C).

### Perfusion fixation and tissue handling

Animals used for the studies of infarct volume and NPC proliferation one week after surgery were transcardially perfusion fixed in deep pentobarbital anesthesia (Mebumal SAD, 50 mg/ml). Ice cold isotonic saline perfusion for two minutes was followed by eight minutes of 4% paraformaldehyde perfusion at a flow rate of 20 ml/minute.

After overnight immersion fixation in 4% paraformaldehyde at 4°C the brains were stored in phosphate buffered saline (PBS) until cryosectioning on a calibrated Leica cryostat (Leica, Germany). The brains were stored in 30% sucrose at 4°C for cryoprotection at least three days prior to tissue sectioning. The brain was mounted with TissueTek^® ^(Sakura Finetek Europe B.V., Zoeterwoude, Netherlands) and cut in the coronal plane. The section thickness was 60 μm. All sections were collected with a random beginning at the level of the anterior commisure. Approximately 200 to 240 sections were collected from one brain and divided into ten series. Each series contained sections with an intersection distance of 600 μm and were sampled in 24-well plates containing anti-freezing solution composed of 30% glycerol, 30% ethylene-glycol, and PBS. The brain sections were stored at -20°C until the final immunohistochemical processing.

### Immunohistochemistry (IHC)

All IHC was carried out as free floating reactions in a specially designed tray system that allowed us to process series from twenty brains at one time. Primary antibodies directed against activated microglia (ED-1), BrdU, COX-2, and neuronal nuclei (NeuN) were used (Table 2). We made double stains for BrdU/ED-1 and NeuN/COX-2. Negative controls included omitting either the primary or secondary antibodies.

**Table 2:** Primary antibodies used for immunohistochemistry.

**Antibody**	**Target**	**Manufacture**	**Working dilution**
**BrdU**	5-bromo-2'-deoxy-uridine	Becton Dickinson Cat. no. 347580	1:200
**COX-2**	Cyclooxygenase 2 enzyme	Cayman Chemical Company Cat. no. 160126	1:4000
**ED-1**	Glycoprotein of 90–100 kD expressed on the lysomal membrane of activated microglia, macrophages and monocytes.	Chemicon International Cat. no. MAB1435	1:4000
**NeuN**	DNA binding neuron-specific protein	Chemicon International Cat. no. MAB377B	1:1000

The brain sections allocated for BrdU staining underwent a denaturizing pretreatment to visualize the BrdU incorporation into the DNA double strand. Sections were incubated in 50% formamide in 50% 2 × SSC (0.3 mol/L NaCl and 0.03 mol/L sodium citrate) buffer at 65°C for two hours. After rinsing in PBS, the tissue was pretreated in 2N HCl at 37°C for 30 minutes followed by washing in 0.1 M boric acid at pH 8.5 for 10 minutes. Thereafter, all stains followed the same protocol. Endogenous peroxidase activity was blocked with 2% H_2_O_2 _in PBS for 20 minutes. All sections were incubated in 5% normal swine serum (NSS), 1% bovine serum albumin (BSA), and 0.3% Triton X (TX) in PBS for 30 minutes at room temperature to prevent a nonspecific immunoreaction. The brain sections were incubated overnight with the primary antibodies at 4°C in 1% BSA and 0.3% TX in PBS in the mentioned working dilutions (Table 2). For COX-2, biotinylated goat anti-rabbit IgG (1:2000) (Vector Laboratories, Burlingame, CA, USA, cat. no. BA-1000), and for BrdU and ED-1, biotinylated donkey anti-mouse F(ab)_2 _(1:2000) (Jackson ImmunoResearch Laboratories INC., West Grove, PA, USA, cat. no. 715-066-150) were used as secondary antibodies. The primary NeuN antibody was biotinylated and incubation with a secondary antibody was therefore omitted. Finally, brain sections were incubated with avidin-biotin-peroxidase complex (ABC) Elite Standard Kit (Vector Laboratories, Burlingame, CA, USA, cat. no. PK-6100) for one hour at room temperature, before peroxidase development with nickel-enhanced DAB or NovaRed^® ^(Vector Laboratories, Burlingame, CA, USA, cat. no. SK-4100 and SK-4800).

### Stereology

The infarct volume was estimated on NeuN-stained coronal sections using the 2D nucleator and the Cavalieri principle [26-29]. The center of the infarct area was marked manually as origin on the computer screen. CAST^® ^software (Visiopharm, Hørsholm, Denmark) generated systematic random directions for measurements using three test lines. The intersections between the test lines and the infarct boundary were marked on the screen and the computer calculated the area. Finally, the infarct volume from each animal was estimated by adding the infarct areas multiplied with the distance between each section.

We used ED-1 and BrdU double-stained sections for estimation of BrdU-positive cells in the DG of the hippocampus (Figure 9A to 9F). The counting procedure followed the optical fractionator design using an Olympus BX50 light microscope (Olympus, Japan) equipped with a motorized specimen stage, a microcator and a 3-CCD video camera interfaced to a PC via a frame grabber [27]. First the DG was delineated with a 4× objective. An area sampling fraction of 28% of the delineated area was used for the cell counting. The CAST^® ^system created an unbiased counting frame with a 40× objective within the delineated DG area. We counted on average 100–150 BrdU positive cells in seven to nine different coronal sections per DG. The whole section thickness of 60 μm was used following analysis of a z-axis distribution.

### Statistics

All statistical analyses were carried out with Stata Intercooled 8.2 software (StataCorp LP, College Station, TX, USA). Physiologic parameters, ADC and BrdU data were analyzed with a one-way ANOVA test with Bonferroni post-hoc analysis for comparison between groups. The infarct volumes were analyzed with an unpaired Student's t-test. P < 0.05 was considered statistical significant.

## Results

### Physiological parameters

All parameters monitored before, during, and after surgery are presented in Table 3. We found a significantly lower pCO_2 _after surgery in the sham group subjected to saline treatment. The blood glucose level was significantly elevated in the sham groups. However, all differences among the four groups are considered of no physiological importance. In general, we found a high blood glucose level in all animals, which could be due to surgical stress or a strain characteristic.

**Table 3:** Physiological parameters.

**Group**	**SHR tMCAo, parecoxib **(n = 21)	**SHR tMCAo, saline **(n = 21)	**SHR sham, parecoxib **(n = 8)	**SHR sham, saline **(n = 9)
MABP, before (mmHg)	137.0 ± 18.82	139.2 ± 17.87	152.3 ± 12.14	144.8 ± 12.02
MABP, during (mmHg)	129.3 ± 19.64	131.4 ± 20.39	141.5 ± 12.37	132.6 ± 16.49
MABP, after (mmHg)	111.4 ± 20.36	120.9 ± 24.33	136.8 ± 19.62	135.0 ± 28.29
				
HR, before (BPM)	374 ± 28.6	364 ± 29.2	363 ± 25.3	373 ± 25.9
HR, during (BPM)	372 ± 24.5	379 ± 28.5	359 ± 24.8	368 ± 21.0
HR, after (BPM)	336 ± 28.3	353 ± 25.3	335 ± 25.8	349 ± 27.3
				
Rectal Temp., before (°C)	37.8 ± 0.40	37.6 ± 0.38	37.6 ± 0.22	37.4 ± 0.30
Rectal Temp., during (°C)	37.8 ± 0.15	37.7 ± 0.14	37.7 ± 0.07	37.7 ± 0.11
Rectal Temp., after (°C)	37.5 ± 0.27	37.6 ± 0.24	37.3 ± 0.18	37.4 ± 0.25
				
pH, before	7.46 ± 0.04	7.45 ± 0.03	7.46 ± 0.02	7.44 ± 0.02
pH, during	7.44 ± 0.02	7.44 ± 0.02	7.45 ± 0.01	7.45 ± 0.02
pH, after	7.43 ± 0.02	7.42 ± 0.02	7.44 ± 0.02	7.44 ± 0.03
				
pCO_2_, before (kPa)	5.70 ± 0.65	5.71 ± 0.58	5.81 ± 0.42	5.92 ± 0.28
pCO_2_, during (kPa)	5.53 ± 0.33	5.52 ± 0.35	5.37 ± 0.22	5.21 ± 0.17
pCO_2_, after (kPa)	5.55 ± 0.21	5.47 ± 0.43	5.20 ± 0.33	5.12 ± 0.22*
				
pO_2_, before (kPa)	28.36 ± 5.01	29.13 ± 3.68	29.17 ± 2.65	29.87 ± 1.65
pO_2_, during (kPa)	29.10 ± 4.24	27.76 ± 4.36	28.49 ± 3.11	27.42 ± 4.17
pO_2_, after (kPa)	29.27 ± 3.47	29.20 ± 2.92	28.75 ± 2.80	28.53 ± 3.29
				
Hemoglobin, before (mmol/L)	9.5 ± 0.42	9.5 ± 0.41	9.5 ± 0.46	9.7 ± 0.77
Hemoglobin, during (mmol/L)	8.6 ± 0.45	8.7 ± 0.41	8.9 ± 0.47	8.8 ± 0.50
Hemoglobin, after (mmol/L)	8.1 ± 0.47	8.1 ± 0.51	8.3 ± 0.39	8.2 ± 0.56
				
Glucose, before (mmol/L)	12.7 ± 2.72	13.0 ± 2.98	15.8 ± 1.45*	15.8 ± 0.77*
Glucose, during (mmol/L)	10.0 ± 2.74	10.3 ± 2.68	13.1 ± 0.78*	12.0 ± 1.62
Glucose, after (mmol/L)	9.8 ± 2.70	10.3 ± 2.88	13.1 ± 2.28*	11.9 ± 1.44
				
Body weight (grams)	314.9 ± 16.74	309.1 ± 20.74	300.5 ± 16.82	294.1 ± 22.39
				
Duration of anesthesia (min)	154.2 ± 6.94	156.9 ± 17.72	154.8 ± 7.48	156.2 ± 9.01

Physiological parameters monitored before, during and after tMCAo or sham procedure in all four groups. Mean values ± SD. One-way ANOVA with Bonferroni post hoc analysis was used for the comparison between the groups. * indicate p < 0.05.

As shown in Figure 2A, animals had a striking weight loss in the first four days postoperatively, regardless of whether the animals belonged to the tMCAo or the sham groups, or whether they were subjected to saline or parecoxib treatment. A similar weight loss profile in the intraluminal tMCAo model has recently been addressed [30,31]. It seems likely that varying degrees of ischemia in the right jaw muscles could contribute to the pronounced decrease in body weight. In three out of 23 animals undergoing MRI 24 hours after surgery, we found enhancement in the DWI signal of the right temporal muscle (Figure 2B).

### MRI

**Figure 3:**
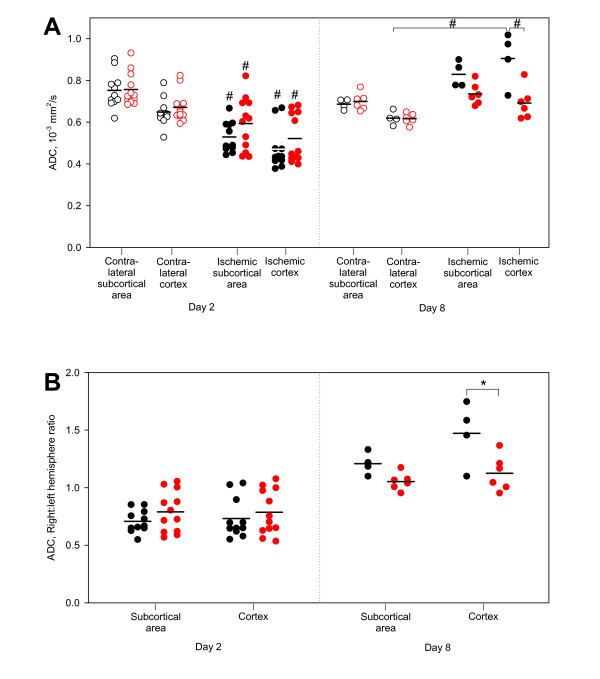
**ADC values and ratios on Day 2 and Day 8 after tMCAo**. **3A **illustrates the absolute ADC values for each of the tMCAo animals that underwent MRI on Day 2 and Day 8. The measurements were performed on cortical and subcortical regions on one coronal ADC map from each animal. Black ○●: tMCAo + saline; and red ○●: tMCAo + parecoxib. The contralateral non-ischemic hemispheres are marked with unfilled symbols, whereas the ischemic hemispheres are represented with filled dots. On Day 2 there was a significant mean ADC decrease in cortex and striatum of both groups (p < 0.01). On Day 8 the ADCs were "pseudonormalized" in the ischemic hemispheres due to cystic brain tissue necrosis. We found a significant mean ADC increase in the cortex of the saline-treated group (p < 0.01) compared with both the contralateral cortex and the ischemic cortex of the parecoxib group. **3B **visualizes the ADC ratios of the ischemic vs. the contralateral hemispheres for both groups on Day 2 and Day 8. Note that the ADC ratios on Day 2 are lower than one in striatum and cortex in both groups which represent the initial ADC decrease after ischemia. However, on Day 8 the ADC ratios lie around or above one due to the "pseudonormalization" phenomenon (p < 0.03). In the cortex of saline-treated animals we found a significantly higher mean ADC ratio. Black ●: tMCAo + saline; and red ●: tMCAo + parecoxib. Mean values are marked with horizontal black lines. # indicates p < 0.01 and * p < 0.03.

Twenty-three tMCAo animals divided equally into two groups receiving either parecoxib or saline treatment underwent DWI and T_2_WI 18–19 hours after the surgical procedures were accomplished (Table 4). The absolute ADC values in the cortex and the subcortical area of both hemispheres from each animal are shown in Figure 3A, and the ADC ratios of the ischemic vs. the contralateral hemisphere are shown in Figure 3B.

**Table 4:** Mean times ± SD for DWI and qRT-PCR studies after surgery.

**Group**	**SHR tMCAo, parecoxib**	**SHR tMCAo, saline**	**SHR sham, parecoxib**	**SHR sham, saline**
**DWI – Day 2**	18 h 57 min ± 3 h 23 min (n = 12)	18 h 39 min ± 2 h 28 min (n = 11)	-	-
**qRT-PCR – Day 2**	25 h 11 min ± 1 h 48 min (n = 6)	23 h 17 min ± 1 h 54 min (n = 6)	25 h 34 min ± 23 min (n = 3)	24 h 36 min ± 45 min (n = 4)

The ADC in the striatum of the non-ischemic hemisphere tended to be slightly higher than in the cortex (Figure 3A). However, this regional difference did not reach statistical significance. We found a significant decrease (p < 0.01) in the ADC value in both striatum and cortex on Day 2 after surgery in both groups (Figure 3B). The mean ADCs were lower in the saline-treated group (approximately 72% of the contralateral hemispheres) than in the parecoxib-treated group (approximately 79% of the contralateral hemispheres) (Figure 3B), but the differences were not significant. Interestingly, we found a clear division of the ADC values in the ischemic cortex of the parecoxib-treated animals. Thus, the animals apparently segregated into a group with low ADCs and a group with high ADCs.

Ten out of the 23 animals scanned on Day 2 underwent similar MRI sequences on Day 8 after surgery. The ADC values were higher in the ischemic than in the contralateral hemisphere in both treatment groups. The mean cortical ADC values were 113% in the parecoxib group compared with 147% in the saline group (p < 0.03), which suggests delayed "pseudonormalization" within the treated animals (see Discussion) [32,33]. This pattern was similar in the subcortical area; however, the difference between the groups was less pronounced (105% of the contralateral hemispheres in the parecoxib group, 121% in the saline group – Figure 3B). A visible infarct on T_2_WI and a "pseudonormal" ADC suggests development of vasogenic brain edema. We found T_2_-weighted infarct changes and high ADCs on Day 8 in three out of four saline-treated animals and two out of six parecoxib-treated rats.

### Cytokine expression

**Figure 4:**
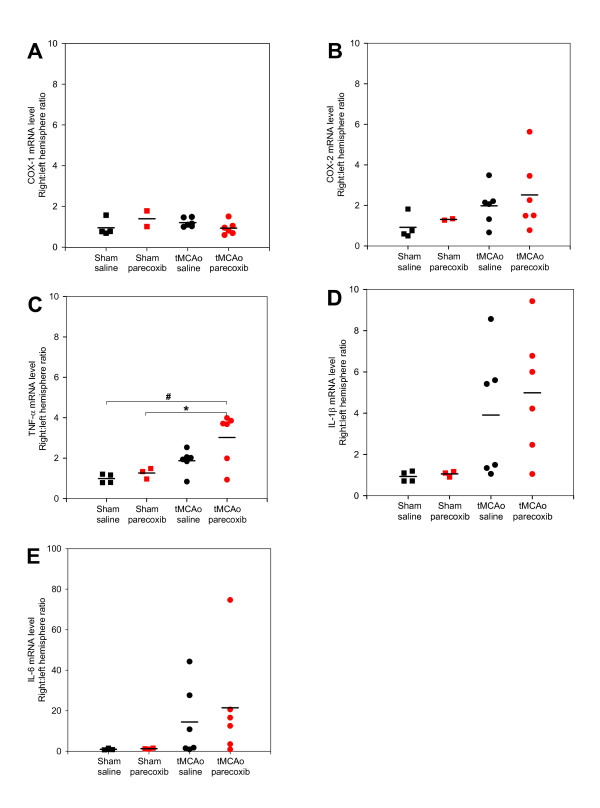
**Cytokine mRNA expression 24 hours after surgery**. The mRNA was purified from each hemisphere separately 24 hours after the end of surgery. The mRNA expression of COX-1, COX-2, TNF-α, IL-1β, and IL-6 is presented as right:left hemisphere ratios for each animal. As expected, we found no indications of COX-1 mRNA upregulation 24 hours after tMCAo (**A**). For COX-2 (**B**), TNF-α (**C**), IL-1β (**D**), and IL-6 (**E**) we found consistent results implying mRNA up-regulation 24 hours after sham or tMCAo. We only found significant TNF-α up-regulation in the parecoxib group due to small sample sizes and large biological variation in the ischemia groups. Note that the COX-1 and COX-2 mRNA ratios were only determined for two animals in the parecoxib sham group. Black ■: sham + saline; red ■: sham + parecoxib; black ●: tMCAo + saline; and red ●: tMCAo + parecoxib. Mean values are indicated with black horizontal lines. # means p < 0.01 and * p < 0.05.

All animals were euthanized within 23–25 hours after the sham or tMCAo procedures ended (Table 4). The cytokine levels were corrected for the expression of the house-keeping gene β-actin and are presented as right:left hemisphere ratios (Figure 4).

**Figure 5:**
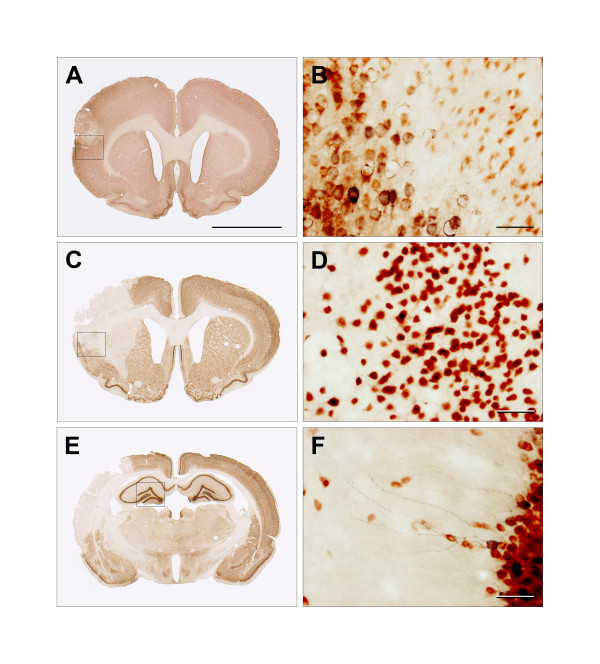
**COX-2 and NeuN double stains 24 hours and one week after tMCAo**. The COX-2 IHC was developed with nickel-enhanced DAB (black), whereas NeuN was visualized with NovaRed^® ^(brownish red). The images **5A **and **5B **are obtained from a pilot study where the animal was euthanized 24 hours after tMCAo. **5A **visualizes a relatively small neocortical infarct in the right hemisphere. The box delineates a part of the ischemic border zone that is shown at forty times magnification in **5B**. The penumbra contains large swollen neurons that express the membrane-bound COX-2 enzyme. In the infarct core the neurons tend to be small and star-shaped due to irreversible neuronal death. **5C **and **5E **are from a saline-treated animal one week after tMCAo. Forty times magnifications of the boxes are shown in **5D **and **5F**. The neurons in the border zone on Day 8 after ischemic injury showed a perinuclear expression pattern of the COX-2 enzyme (**5D**). COX-2^+ ^neurons can be found in areas like the neocortex, piriform cortex and the DG of the hippocampus under normal conditions. **5F **shows COX-2 expressed in dendrites of neurons in the molecular cell layer of the DG. The scale bar in **5A **is 5 mm, whereas the scale bars in **5B**, **5D **and **5F **equals 50 μm.

As shown in Figure 4A, the COX-1 mRNA expression was not affected by transient focal brain ischemia. On the contrary, we found clear indications of a higher COX-2 mRNA level 24 hours after ischemia (Figure 4B). The COX-2 up-regulation happened regardless of whether the animals underwent saline or parecoxib treatment. In immunohistochemical pilot studies we found a consistent COX-2 protein presence in the border zone of the infarct 24 hours after tMCAo (Figure 5A and 5B). On Day 8 after ischemia it was impossible to visualize the same COX-2 protein expression around the matured infarct (Figure 5C and 5D). Together with IL-1β and IL-6, TNF-α is one of the major pro-inflammatory cytokines released by activated microglia following ischemic brain injury. For all three cytokines, we saw a similar mRNA expression pattern 24 hours after tMCAo (Figure 4C, 4D, and 4E). In the two ischemia groups our measurements indicated an mRNA up-regulation of TNF-α, IL-1β, and IL-6 that was unaffected by COX-2 enzyme blockage. For TNF-α, the mRNA up-regulation differed significantly between the parecoxib-treated tMCAo group and the two sham groups. The significant differences in TNF-α expression should be interpreted with caution due to large spreads in small sample sizes.

### Infarct volume

**Figure 6:**
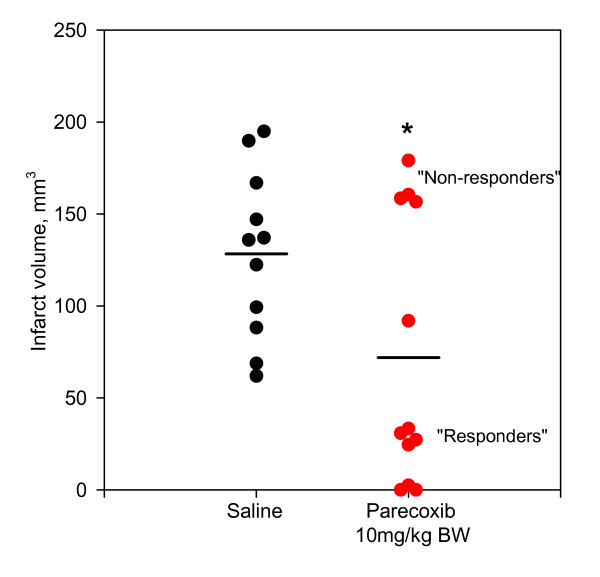
**Infarct volume one week after tMCAo**. The infarct volume was estimated by means of the 2D nucleator and the Cavalieri principle applied on NeuN stained brain sections one week after surgery. Saline and parecoxib-treated animals are marked with black and red dots (●) respectively. Parecoxib significantly reduced the mean infarct size (p < 0.03). Interestingly, the parecoxib group was divided into two subgroups suggesting a "responder" and "non-responder" phenomenon of the COX-2 inhibitor. All eleven animals in the saline group had an infarction pattern with neocortical involvement. In the parecoxib group only five out of twelve animals had cortical infarction. Mean values are marked as black horizontal lines. * indicate p < 0.03.

**Figure 7:**
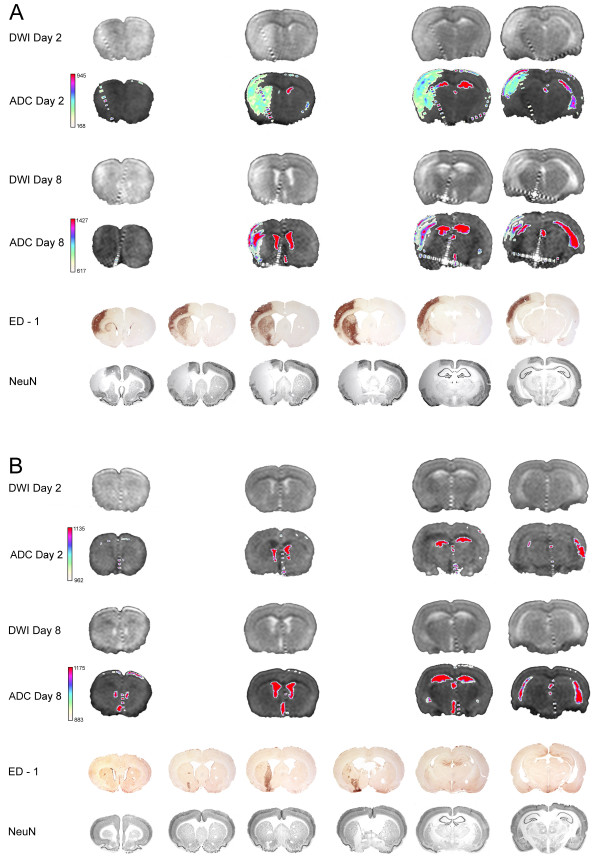
**Examples of saline and parecoxib treatment one week after tMCAo**. Representative examples of saline (**A**) and parecoxib (**B**) treatment are shown. The first four rows show DWI and the corresponding ADC maps from Day 2 and 8. Note the "pseudonormalization" of the ADC map in the saline-treated animal on Day 8 (**A**). The last two rows show the ED-1 and NeuN stains. The area where activated microglia and invading white blood cells are seen on the ED-1 stain clearly overlap the area of neuronal loss visualized on the NeuN stain. The treatment effect of parecoxib was only seen in the right MCA area whereas the medial striatal area supplied by the AChA did not respond.

Estimation of the total infarct volume using the 2D nucleator and the Cavalieri principle on NeuN-stained sections one week after tMCAo showed that the parecoxib-treated rats fell into two subgroups. In seven out of the twelve rats subjected to parecoxib treatment, we found small subcortical infarcts restricted to the territory of the right anterior choroidal artery (AChA) (Figure 7). The AChA can be considered an end artery due to the variation in collateral blood supply from the MCA and posterior cerebral artery (PCA) [34]. In the remaining five rats in the parecoxib group, we found relatively large infarcts involving the lateral aspect of the right striatum and varying parts of the overlaying neocortex. This difference in infarct pattern in the parecoxib group suggests a divided response like a responder vs. non-responder phenomenon. In all eleven saline-treated tMCAo rats, we found a classical MCA infarct pattern comprising most of the striatum and varying parts of the temporal and parietal neocortex. Overall, the parecoxib treatment reduced the mean infarct volume significantly (p < 0.03) (Figure 6).

### Neuronal precursor cell proliferation in the molecular layer of the dentate gyrus

**Figure 8:**
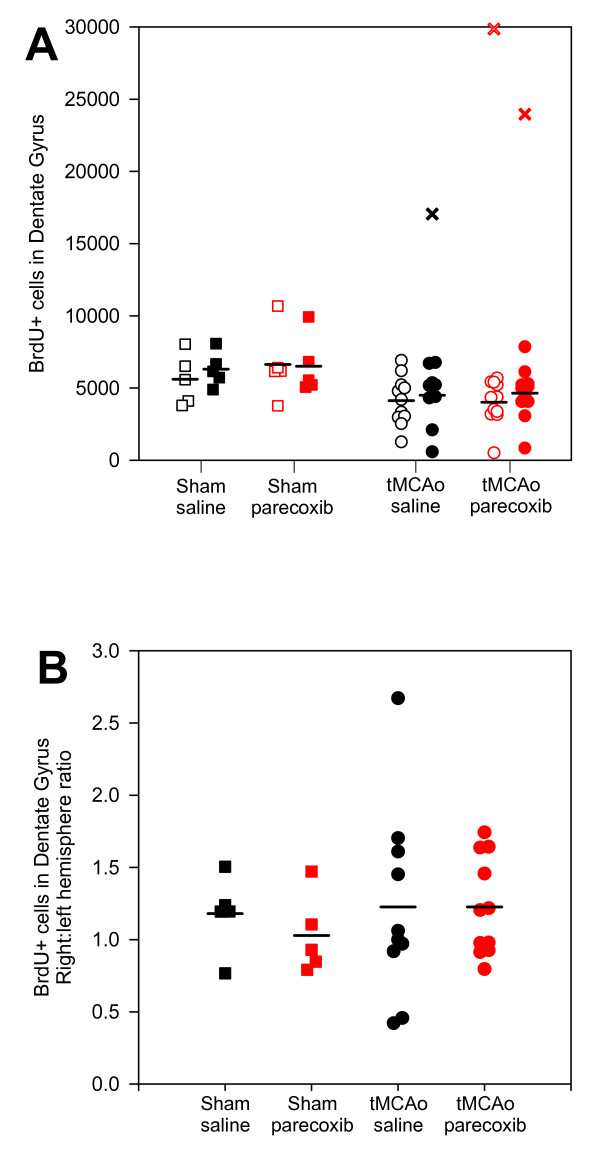
**BrdU incorporation in the dentate gyrus of the hippocampus**. We estimated in average between four to six thousand BrdU-positive cells in the hippocampal DG (**A**). We revealed no significant differences between or within the four groups. The mean number of BrdU-positive cells was generally lower in the tMCAo groups than in the sham groups. However, if the DG was affected by ischemia (see Figure **9D **to **9I**) the BrdU incorporation increased dramatically (black **× **and red **×**). Note that in one animal (marked with red **×**) the ischemic injury also affected the contralateral hippocampus. **B **shows the BrdU incorporation ratio between the right and left hemispheres. The mean ratios for the four groups indicated no difference in the BrdU incorporation between the hemispheres or groups. Black □■: sham + saline; red □■: sham + parecoxib; black ○●: tMCAo + saline; and red ○●: tMCAo + parecoxib. The ischemic or sham (right) hemispheres are represented with filled symbols, whereas the contralateral (left) hemispheres are unfilled. Mean values are indicated with black horizontal bars.

**Figure 9:**
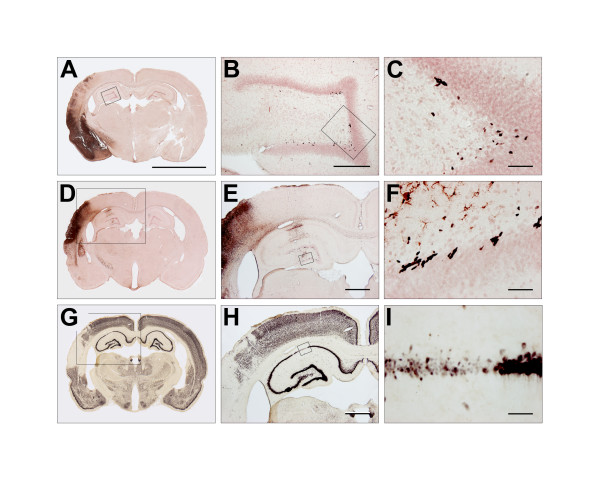
**BrdU, ED-1 and NeuN stains one week after tMCAo**. **9A **to **9F **show the IHC for BrdU and ED-1. BrdU was developed with nickel-enhanced DAB (black), whereas the activated microglia marker ED-1 was visualized with NovaRed^® ^(brownish red). **9A **shows a representative example of a tMCAo animal one week after surgery. Four and forty time magnifications of the boxes in **9A **and **9B **are shown in **9B **and **9C**, respectively. The BrdU^+ ^cells are typically found in clusters in the subgranular cell layer in DG. Our counting procedure started with delineating the DG. Hereafter, the BrdU^+ ^cells in the whole DG were counted at forty times magnification (**9C**). We generally observed a very scarce ED-1 expression in the DG unless the hippocampus was directly affected by ischemic injury. **9D **to **9I **show an example of ischemia affecting the right hippocampus. The boxed area in **9D **is shown at higher magnification in **9E**. Activated microglia was abundantly seen in a part of the CA-1 and the whole DG. **9F **shows a forty time magnification of the box in **9E**. Clearly, activated microglia had an intimate relation to an increasing number of BrdU^+ ^cells. Panel **9G **to **9I **show NeuN IHC developed with nickel-enhanced DAB. **9G **and **9H **correspond to **9D **and **9E**. The boxed area in **9H **of the CA-1 is shown at forty time magnification in **9I**. Note the ischemic degeneration of this part of the CA-1. The scale bar in **9A **is 5 mm. In **9B **the scale bar represents 300 mm, and in **9E **and **9H **1 mm. The scale bars in **9C**, **9F **and **9I **equals 50 μm.

BrdU incorporation in the DG of the hippocampus was unaffected by ischemia or parecoxib treatment as shown in Figure 8A. The hippocampus is usually not affected by ischemia after tMCAo. However, in two animals with large stroke volumes (marked with crosses in Figure 8A), we saw ischemic damage of the DG and infiltration with activated microglia and macrophages (Figure 9D to 9I). We excluded the BrdU counts from these animals in our statistical analyses. However, their impact on the mean BrdU number would not change the stated conclusions.

The right:left hemisphere ratio is illustrated in Figure 8B. We observed no difference in BrdU incorporation between the ischemic and contralateral hemispheres.

## Discussion

The aims of the present study were to investigate different effects of parecoxib at a clinically relevant dosage in a model of transient focal brain ischemia. However, this study cannot be considered a dose-response study following the STAIR criteria [35]. The most significant finding is the reported mean stroke volume reduction in SHRs treated with parecoxib IP after 90 minutes of tMCAo. The post-ischemic ADC increase in the neocortex due to "pseudonormalization" was consistently and significantly lower in parecoxib-treated than in the saline-treated animals.

We used out-bred male SHR rats in the current study owing to our unpublished experience with the success rate of the intraluminal tMCAo in different rat strains. In addition, hypertension is one of the most prominent risk factor in the underlying pathophysiology of the ischemic stroke [36]. Monitoring of the relative decrease in the blood flow in the ischemic MCA territory during surgery has an unmistakable relevance in the intraluminal filament occlusion model [37,38]. Recent pilot studies conducted in our lab with laser-doppler blood flow measurements have confirmed a decrease in the relative blood flow during tMCAo. However, since this study was performed without peroperative laser-doppler flow monitoring, we used the ED-1 immunohistochemical stain as a histological exclusion criterion. Activated microglia is known to be a very sensitive marker for different kinds of central nervous system (CNS) injury [39,40]. One animal subjected to tMCAo was excluded from our study because of lacking ED-1 positivity in the ischemic hemisphere due to incomplete occlusion of the MCA origin. Subarachnoid hemorrhage is another well-described pitfall in the intraluminal tMCAo model [37,38]. We observed one animal with subarachnoid hemorrhage. In addition, we lost three tMCAo animals due to unexpected deaths, but without any macroscopic signs of intra-cerebral or subarachnoid hemorrhages.

### Pro-inflammatory cytokine mRNA levels unaffected by parecoxib treatment

We decided to investigate the mRNA expression of major pro-inflammatory cytokines 24 hours after surgery since the expression of most pro- and anti-apoptotic proteins peaks 12–36 hours after ischemic brain injury [41]. The RNA was purified from the whole hemispheres since the injury induced by the intraluminal tMCAo model affects both cortical and subcortical territories. Many of our findings are supported by previous reports [2,42,43] and additional immunohistochemical observations.

However, we were not able to show an effect of parecoxib administration due to the divided treatment response of parecoxib, large biological variation in stroke volume, and small sample sizes. It is evident that dynamic changes in mRNA expression are missed since only a single time point was selected for our measurements of mRNA levels by qRT-PCR at 24 hours. Further, the blockage of the COX-2 enzyme does not imply that the transcription of pro-inflammatory cytokines is affected.

### Neuroprotective effect measured on apparent diffusion coefficient values

Diffusion weighted magnetic resonance imaging is a very sensitive method in the detection of early ischemic injury of cerebral tissue in animal models of focal ischemia as well as in humans [44,45]. DWI provides information about the self-diffusion of water and allows detection of ischemic injury within a few minutes after regional perfusion is decreased [46]. The technique has enjoyed wide use in neuroprotective animal studies as a valuable measure of lesion size and the extent of cytotoxic edema [47,48].

We observed a beneficial effect of parecoxib administration based on ADC measurements, as hyperacute ADC reduction was less pronounced in parecoxib-treated than in saline-treated animals. This reduction immediately after the decrease in regional perfusion is believed to be caused by a shift of water from the extracellular to the intracellular space due to cytotoxic edema [49]. Others have also demonstrated less ADC reduction in early focal ischemia after neuroprotective therapy in experimental animal studies [48]. Furthermore, we observed a beneficial effect of parecoxib administration based on cortical ADC measurements obtained one week after surgery. In rodents, ADC begins to normalize 24 to 48 hours after onset of ischemia ("pseudonormalization") due to progressive extracellular edema, which reflects vasogenic edema and a subsequent increased diffusion [50]. In the following days, ADC increased up to 300% of normal values, as cell lysis caused increased water diffusion in the necrotic stroke cavity. In the present study, the markedly lower "pseudonormalized" cortical ADC in the parecoxib-treated group therefore reflects a lesser degree of infarct formation one week after the ischemic injury. ADC changes can therefore be considered a measure of the severity of the ischemic stroke [51]. We found indications of a significant correlation between the ADC decrease and the IL-1β mRNA level after 24 hours (data not shown). As proposed by Mancuso et al. [52], we believe that the initial ADC decrease can be linked to the degree of neuroinflammation following tMCAo.

One of the limitations in the MRI part of our study is the slice thickness of two millimeters. Hence, relatively small infarcts can hardly be detected as was the case for a number of animals in the parecoxib-treated group (Figure 7B). We therefore were not able to calculate the stroke volume based on either DWI or T_2_WI. Our ADC data were obtained without baseline lesion size measurements prior to drug administration. The efficacy of parecoxib treatment presented here may therefore encompass pretreatment bias [53].

### Neuronal precursor cell proliferation is not affected by parecoxib treatment

A large number of factors including age, environmental enrichment, exercise, growth, and neurotrophic substances influence neurogenesis in the adult brain [20-23]. Kumihashi et al. [54] were the first to address the possible role of COX-2 in neurogenesis after transient forebrain ischemia in gerbils. They found a significant decrease in neurogenesis in DG two weeks after the insult in animals treated with acetylsalicylic acid (30 mg/kg BW). Sasaki et al. [55,56] used a model of transient forebrain ischemia in COX-2 knock-out and wild type mice to investigate the role of the COX-2 protein in post-ischemic hippocampal neurogenesis. Ten days after the ischemic insult, indomethacin (10 mg/kg BW) and the selective COX-2 inhibitor NS398 (20 mg/kg BW) significantly reduced BrdU incorporation in the DG of wild type mice. A similar decrease in hippocampal NPC proliferation was found in COX-2 knock-outs. Recently, Kluska et al. [57] published an interesting study where BrdU incorporation in the DG was evaluated up to ten weeks after photothrombotic cortical stroke in Wistar rats. Although the total number of BrdU^+ ^cells decreased over time, there was a significant increase in BrdU^+ ^cells with a mature NeuN phenotype. Treatment with MK-801 (2 mg/kg BW) and indomethacin (2.5 mg/kg BW) enhanced neurogenesis in the DG four weeks after photothrombotic ischemia [57]. We found no increase in BrdU incorporation in the DG in the ischemic hemispheres after tMCAo. Hoehn et al. [58] recently reported a suppression of BrdU^+ ^cells in the subventricular zone (SVZ) within the first week after reperfusion following tMCAo in Sprague-Dawley rats. In the same paper enhanced neurogenesis in the striatum and cortex due to indomethacin intake (2.5 mg/kg BW) was observed 14 and 28 days after the ischemic insult.

As shown in Figure 9D to 9I ischemic damage of CA1 in the hippocampus dramatically stimulated the BrdU uptake. Although the DG was not directly affected by the ischemic injury, the presence of ED-1^+ ^microglia indicated a neuroinflammatory activity. Ekdahl et al. [59] and Monje et al. [60] reported that inflammation observed after lipopolysaccharide (LPS) administration in rat models of status epilepticus and whole brain irradiation has detrimental effects on neurogenesis in the adult brain. Further blockage of microglia activation with minocycline restored hippocampal neurogenesis after LPS-induced neuroinflammation [59,60]. We think that microglia activation after different kinds of brain injury should not be considered a homogeneous response. This notion is supported by a recent *in vitro *study where different microglia activation types determined whether the effect on NPCs was beneficial or detrimental [61]. Further microglia can stimulate hippocampal neurogenesis under non-pathological conditions [62].

### Different responsiveness to parecoxib treatment

Surprisingly, we found a divided response in animals treated with parecoxib IP twice daily for one week. Seven out of twelve animals had small subcortical infarcts, whereas the last five animals had large stroke volumes involving substantial parts of the neocortex. Over the past five years, both Iadecola et al. [2-4,8] and Candelario-Jalil et al. [7,9,10] have reported consistent neuroprotective effects of the selective COX-2 inhibitor nimesulide in different rodent models of ischemic brain injury. However, none of the mentioned studies observed a divided treatment effect similar to the one observed in our study. We continued the parecoxib administration beyond the maturation point of ischemic brain injury. A possible explanation for the observed divided response could therefore be secondary thrombosis of the MCA origin due to endothelial damage by the occluding filament. This hypothesis is supported by the fact that selective COX-2 inhibitors impair the delicate endothelial balance of COX-1 dependent thromboxane A_2 _(TXA_2_) and COX-2 dependent prostacyclin (PGI_2_) [14,15]. Accumulation of TXA_2 _favors platelet aggregation, vasoconstriction, and smooth muscle cell proliferation.

New studies are necessary to elucidate whether the observed treatment response of parecoxib is due to a rat strain characteristic, a dose-response relation or the way of drug administration.

## Conclusion

IP parecoxib administration (10 mg/kg) during tMCAo was neuroprotective as evidenced by a large reduction in mean infarct volume and cortical ADC measurements one week after tMCAo. Increased pro-inflammatory cytokine levels measured after 24 hours remained unaffected. Hippocampal granule cell BrdU incorporation one week after tMCAo as a measure for post-injury NPC proliferation was not affected by parecoxib administration. The presence of ED-1^+ ^activated microglia in the hippocampus was related to an increase in BrdU uptake in the DG.

## Abbreviations

**ABC**: Avidin-Biotin-peroxidase Complex; **AChA**: Anterior Choroidal Artery; **ADC**: Apparent Diffusion Coefficient; **BBB**: Blood Brain Barrier; **BPM**: Beats Per Minute; **BrdU**: 5-bromo-2'-deoxy-uridine; **BSA**: Bovine Serum Albumin; **BW**: Body Weight; **CCA**: Common Carotid Artery; **cDNA**: complimentary DeoxyriboNucleic Acid; **CNS**: Central Nervous System; **COX-1**: CycloOXygenase 1; **COX-2**: CycloOXygenase 2; **DAB**: 3,3'-DiAminoBenzidine; **DG**: Dentate Gyrus; **DWI**: Diffusion Weighted Imaging; **ECA**: External Carotid Artery; **FA**: Femoral Artery; **Hb**: Hemoglobin; **HR**: Heart Rate; **ICA**: Internal Carotid Artery; **IHC**: ImmunoHistoChemistry; **IL**: InterLeukin; **IM**: IntraMuscular; **IP**: IntraPeritoneal; **IV**: IntraVenous; **LA**: Lingual Artery; **LPS**: LipoPolySaccharide; **MA**: Maxillary Artery; **MABP**: Mean Arterial Blood Pressure; **MCA**: Middle Cerebral Artery; **mRNA**: messenger RiboNucleic Acid; **MRI**: Magnetic Resonance Imaging; **NeuN**: Neuronal Nuclei; **NPC**: Neuronal Precursor Cell; **N_2_O**: Nitrous Oxide; **NSS**: Normal Swine Serum; **OA**: Occipital Artery; **O_2_**: Oxygen; **PA**: Pterygopalatine Artery; **PBS**: Phosphate Buffered Saline; **PCA**: Posterior Cerebral Artery; **PGI_2_**: Prostacyclin I_2_; **qRT-PCR**: quantitative Reverse Transcriptase Polymerase Chain Reaction; **SAH**: SubArachnoid Hemorrhage; **SHRs**: Spontaneously Hypertensive Rats; **STA**: Superior Thyroid Artery; **SVZ**: SubVentricular Zone; **tMCAo**: transient Middle Cerebral Artery occlusion; **TNF-α**: Tumor Necrosis Factor Alpha; **T_2_WI**: T_2 _Weighted Imaging; **TX**: Triton X; **TXA_2_**: ThromboXane A_2_.

## Competing interests

The author(s) declare that they have no Competing interests.

## Authors' contributions

JK designed the study, performed all animal experiments and drug administration, participated in MRI, did all tissue sectioning, staining, mounting and counting, analyzed data, and wrote the paper. KK participated in study design, purified mRNA from the brain samples, performed qRT-PCR, and analyzed qRT-PCR data. GC did the MRI. MP made the ADC maps, and contributed to MR data analysis. LR advised on the MR studies and the MRI analysis. JF and SN helped draft the manuscript. JRN advised in the use of stereologic tools, helped in statistical analyses and data interpretation. LCBR helped designing the study, provided lab facilities, helped to interpret data, and drafted the manuscript. All authors read and approved the final manuscript.
